# Inflammatory Profiles of Tracheal Biopsies From SARS-CoV-2 Patients

**DOI:** 10.3389/fmicb.2022.851460

**Published:** 2022-03-16

**Authors:** Giacomo Fiacchini, Agnese Proietti, Anello Marcello Poma, Miriana Picariello, Iacopo Dallan, Fabio Guarracino, Francesco Forfori, Gabriella Fontanini, Luca Bruschini

**Affiliations:** ^1^Otolaryngology, Audiology and Phoniatric Operative Unit, Department of Surgical, Medical and Molecular Pathology and Critical Care Medicine, University of Pisa, Pisa, Italy; ^2^Unit of Pathological Anatomy, University Hospital of Pisa, Pisa, Italy; ^3^Department of Surgical, Medical, Molecular Pathology and Critical Area, University of Pisa, Pisa, Italy; ^4^Cardiothoracic and Vascular Anaesthesia and Intensive Care, Department of Anaesthesia and Critical Care Medicine, Azienda Ospedaliero-Universitaria Pisana (AOUP), Pisa, Italy

**Keywords:** COVID-19, SARS-CoV-2, virus replication, tracheal lesions, prolonged mechanical ventilation

## Abstract

**Purpose:**

An increasing number of laryngotracheal complications in mechanically ventilated COVID-19 patients has been reported in the last few months. Many etiopathogenetic hypotheses were proposed but no clear explanation of these complications was identified. In this paper we evaluated the possibility that the tracheal mucosa could be a high viral replication site that could weaken the epithelium itself.

**Methods:**

Subjects for the COVID-19 group and the control group were selected retrospectively according to specific criteria. Patients’ basic and clinical data were recorded and analyzed. Tracheal samples of both groups were collected during surgical tracheostomies and then analyzed from a histological and genetic-transcriptional point of view.

**Results:**

Four COVID-19 patients were enrolled in this study and compared with four non-COVID-19 patients. No laryngotracheal complications were identified in both groups. The SARS-CoV-2 was detected in one out of four COVID-19 samples. A subepithelial inflammatory lymphomonocyte infiltrate was observed in all patients but two cases of the COVID-19 group showed vasculitis of small subepithelial vessels associated with foci of coagulative necrosis. Two gene sets (HALLMARK_INFLAMMATORY_RESPONSE and HALLMARK_ESTROGEN_RESPONSE_LATE) were significantly deregulated in COVID-19 patients compared to the control group.

**Conclusion:**

The altered inflammatory response of the COVID-19 patients could be another possible explanation of the increasing number of laryngotracheal complications.

## Introduction

The coronavirus disease 2019 (COVID-19) outbreak has led to a significant and unprecedent increase in laryngotracheal complications and their potential life-threatening sequelae in patients subjected to invasive mechanical ventilation (Fiacchini et al., 2021; Piazza et al., 2021; Sandu, 2021). Many etiopathogenetic hypotheses were proposed such as the pronation maneuvers which could increase the cuff pressure on the tracheal walls (Crivello et al., 2022), the microvascular injury of laryngo-tracheal mucosa caused by the prothrombotic and antifibrinolytic state of these patients, the use of high dose systemic steroids or more simply due to unreported mistakes or accidents by physically and emotionally exhausted health care professionals. However, to date, no clear explanation was identified but a list of recommendations were proposed to prevent and manage this type of complication (Meister et al., 2021).

Another possible cause of laryngotracheal lesions could be the high viral replication within the laryngotracheal mucosa which could weaken the epithelium itself. In fact, SARS-CoV-2 particles were observed in tracheal epithelial cells and within the extracellular mucus in the tracheal lumen of trachea samples taken during autopsies (Bradley et al., 2020).

In this work, we investigated this last etiopathogenetic hypothesis by performing a histological and genetic-transcriptional analysis of tracheal samples taken during surgical tracheostomies in critically ill COVID-19 patients subjected to mechanical invasive ventilation and comparing them with tracheal samples taken from non-COVID-19 patients.

## Materials and Methods

Subjects for the COVID-19 group and the control group (non-COVID-19 patients) were selected retrospectively according to the following criteria:

- age from 18 to 75 years;
- admitted to the Intensive Care Units (ICU) of our tertiary referral hospital between November 1 and December 31, 2020 (Italian second wave) and requiring invasive mechanical ventilation for Acute Respiratory Distress Syndrome (ARDS) caused by SARS-CoV-2 (COVID-19 group) or for other pathologies (control group);
- SARS-CoV-2 detected in nasopharyngeal/oropharyngeal swabs (COVID-19 group) or not detected (control group); and
- subjected to open surgical tracheostomy where a small anterior portion of one or two tracheal rings is removed (Cheung and Napolitano, 2014; Brass et al., 2016) and submitted in 4% buffered formalin to the Surgical Pathology Department of our hospital. The decision to perform a percutaneous or an open surgical tracheostomy in COVID-19 patients was taken by the “tracheo-team” (two otolaryngologists and two anesthesiologists) according to internal guidelines derived from the most recent literature (Bassi et al., 2020; Takhar et al., 2020).

Patients’ basic and clinical data such as age, sex, COVID-19 status, comorbidities, duration of invasive mechanical ventilation with oro-tracheal tubes before open surgical tracheostomy, surgical complications and pharmacological treatments were recorded and analyzed. Data on comorbidities were collected using the Adult Comorbidity Evaluation 27 index (ACE-27; Piccirillo et al., 2004). This study was approved by the Local Ethics Committee on June 24, 2021. Written informed consent to collect deidentified data was obtained from all patients. This study followed the Strengthening the Reporting of Observational Studies in Epidemiology (STROBE) reporting guideline.

### Histological Analysis

One paraffin-embedded inclusion was obtained from each biopsy and three-micrometer thick sections were cut from each sample and stained with Hematoxylin–Eosin (Diapath Spa, Bergamo, Italy).

### Immunohistochemistry

Three-micrometer thick sections were cut from each sample, dewaxed, pretreated by cell conditioner at 95°C for 32 min with ULTRA CC1 ready-to-use solution (Ventana Medical Systems, Inc., Oro Valley, AZ, United States), and thereafter incubated with anti-SARS Nucleocapsid Protein Rabbit Polyclonal antibody (Novus Biologicals; dilution 1:500 and at 36°C for 32 min). The antibody–antigen binding has been detected using the OptiView DAB IHC Detection kit (Ventana Medical Systems, Inc., Oro Valley, AZ, United States). Then slides were counterstained with Hematoxylin II and Bluing Reagent (Ventana Medical Systems, Inc., Oro Valley, AZ, United States) for 8 min.

Three-micrometer thick sections were cut from each sample were stained with ready-to-use CONFIRM anti-CD3 (2GV6) Rabbit Monoclonal Primary Antibody, CONFIRM anti-CD20 (L26) Mouse Monoclonal Primary Antibody, CONFIRM anti-CD4 (SP35) Mouse Monoclonal Primary Antibody, CONFIRM anti-CD8 (SP57) Rabbit Monoclonal Primary Antibody, CONFIRM anti-CD68 (KP-1) Mouse Monoclonal Primary Antibody (Roche Diagnostics Ventana Medical Systems, Inc., Oro Valley, AZ, United States), and CONFIRM anti-CD34 (QBEnd/10) Mouse Monoclonal Primary Antibody. The antibody–antigen binding has been detected using the ultraView Universal DAB Detection Kit (Ventana Medical Systems, Inc., Oro Valley, AZ, United States). Staining was done on an automated IHC/ISH slide staining system (BenchMark ULTRA—Ventana Medical Systems, Inc., Oro Valley, AZ, United States).

### Gene Expression Analysis and Detection of SARS-CoV-2

Four unstained 10 μm-thick formalin-fixed paraffin-embedded sections were used for RNA isolation using the RNeasy FFPE kit (Qiagen, Hilden, Germany). RNA quality was tested by spectrophotometry (Xpose, Trinean, Gentbrugge, Belgium). About 150 ng of RNA were used for the RT-PCR assay to detect the SARS-CoV-2 using the Easy SARS-CoV-2 WE kit (Diatech Pharmacogenetics, Jesi, Italy). The assay is designed to target the viral nucleocapsid (N) and RNA-dependent RNA Polymerase (RdRp) genes. Viral assays were run in duplicates. A sample was deemed positive when at least one of the targets was amplified, as suggested by the manufacturer. For the gene expression assay, about 150 ng of RNA were hybridized at 65°C for 21 h with capture and reporter probes of the Human Host Response panel (nanoString Technologies, Seattle, WA, United States). All procedures were performed following the manufacturer’s suggestions.

### Statistical Analysis

Raw expression counts were normalized using the Advanced Analysis module of the nSover v.4.0 (nanoString Technologies, Seattle, WA, United States). Low count genes (raw numbers below 20 counts) were filtered out, and normalized gene expression levels were log2 transformed. Differentially expressed genes (DEG) between COVID-19 cases and controls were computed by a linear model using control samples as baseline and following the procedures of the Advanced Analysis module of the nSolver software. Values of *p* were adjusted with the Benjamini-Hochberg method, and false discovery rates (FDR) below 0.05 were considered significant. The ranked gene list was used for the gene set enrichment analysis (GSEA) following the procedures of the clusterProfiler Bioconductor package v.3.13. In detail, the Hallmark collection was used as reference database (Liberzon et al., 2015), and a minimum gene set size cut-off of 10 genes was set. The immune cell abundances were computed using the method described by Middleton et al. (2020) and compared by the Mann–Whitney *U* test. A value of *p* of 0.05 was set as significance cut-off. The analyses and plots were done in R environment v.4.1.0 (https://www.r-project.org/, last accessed May 31, 2021), unless otherwise specified.

## Results

### General Data Analysis

From November 1 to December 31, 2020, 62 patients were admitted to the COVID-19 dedicated ICU of our hospital. Twenty patients were referred for tracheostomy and the “tracheo-team” decided to perform an open surgical tracheostomy in four of them. All four patients were enrolled in this study and all of them got a positive PCR test result for Sars-CoV-2 performed the day before the surgical procedure. Four control patients matched for age and sex were selected according to our criteria in the aforementioned time frame. Patients’ basic and clinical data are reported in Table 1.

**Table 1:** Patients’ basic and clinical data.

ID patient	Age	Sex	COVID-19 status	Comorbidities (ACE-27)	Main pathology	IOTI (days)	Laryngo-tracheal complications	Outcome	SBP (mmHg)	DBP (mmHg)	HR (bpm)	Antibiotics	Antivirals	Hydroxy chloroquine	Steroids	Low-molecular-weight heparin
1	65	F	+	Mild	ARDS—Covid-19	18	None	Dead	110	60	55	Yes	No	No	Yes (dexamethasone 8 mg daily)	Yes (prophylactic dosage)
2	60	F	+	Moderate	ARDS - Covid-19	17	None	Discharge	110	65	60	Yes	No	No	Yes (dexamethasone 8 mg daily)	Yes (therapeutic dosage)
3	56	F	+	Mild	ARDS - Covid-19	13	None	Discharge	120	65	60	Yes	No	No	Yes (dexamethasone 8 mg daily)	Yes (therapeutic dosage)
4	48	M	+	None	ARDS - Covid-19	14	None	Discharge	120	70	60	Yes	No	No	Yes (dexamethasone 8 mg daily)	Yes (therapeutic dosage)
5	75	F	−	Moderate	Intracranial Hemorrhage	7	None	Dead	120	70	80	Yes	No	No	No	Yes (prophylactic dosage)
6	62	M	−	None	Trauma	6	None	Discharge	135	75	78	Yes	No	No	Yes (dexamethasone 4 mg daily)	Yes (prophylactic dosage)
7	70	F	−	Mild	Complications of Cardiac Surgery	15	None	Discharge	120	90	90	Yes	No	No	Yes (dexamethasone 8 mg daily)	Yes (therapeutic dosage)
8	65	M	−	Moderate	Complications of Thoracic Surgery	14	None	Discharge	110	60	70	Yes	No	No	No	Yes (prophylactic dosage)

*ACE-27, adult comorbidity evaluation 27; ARDS, acute respiratory distress syndrome; DBP, diastolic blood pressure; HR, heart rate; IOTI, invasive oro-tracheal intubation; SBP, systolic blood pressure*.

### Histological Findings

**Figure 1:**
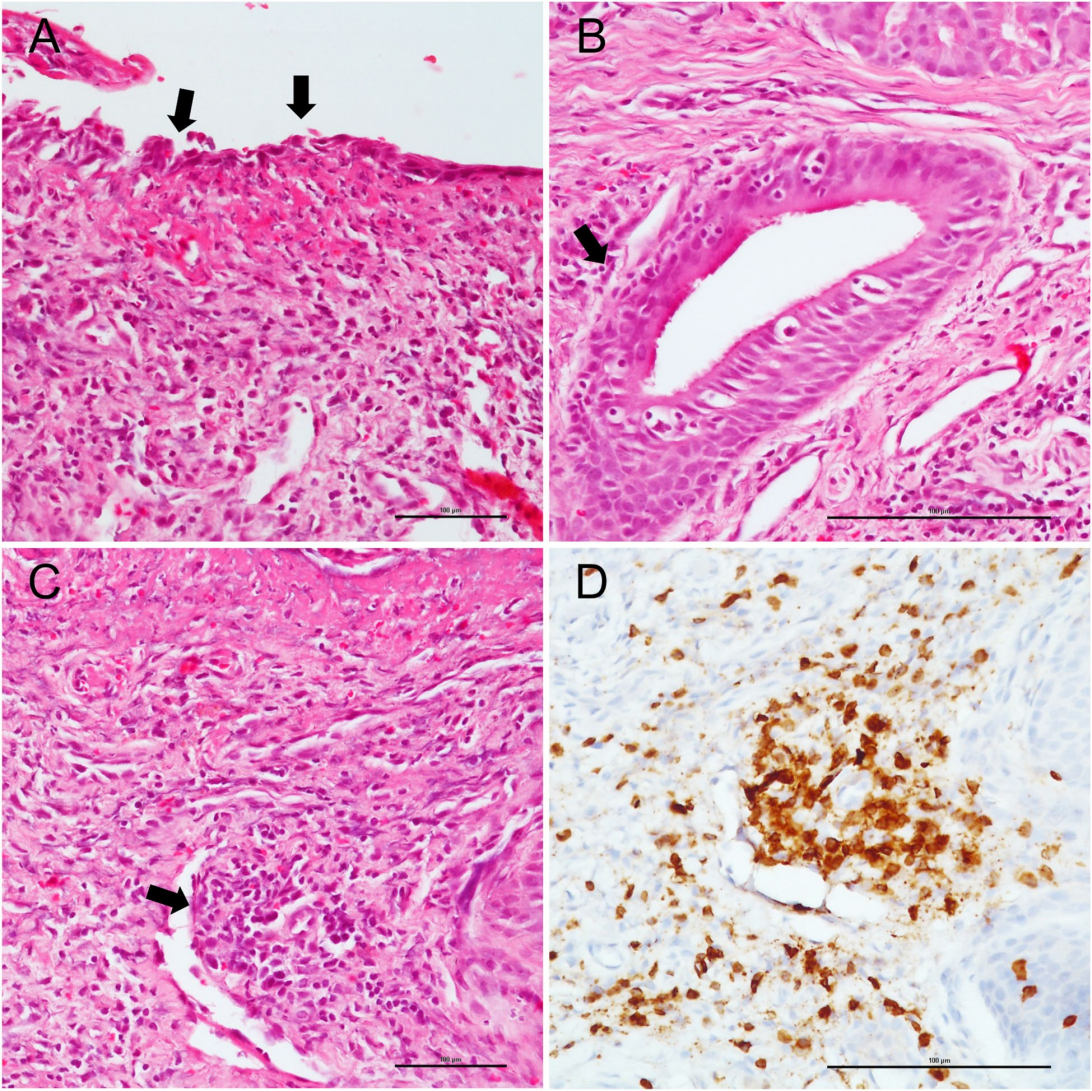
Pathological tracheal alterations in a COVID-19 case. **(A)** epithelial erosion is highlighted by black arrows. **(B)** subepithelial inflammatory lymphomonocyte infiltrate (black arrow). **(C)** hematoxylin and eosin staining shows lymphocytic vasculitis of small subepithelial vessel (black arrow), which is confirmed by CD3 staining. **(D)** Scale bars refer to 100 micrometers.

A predominantly subepithelial inflammatory lymphomonocyte infiltrate was observed in all COVID-19 and non-COVID-19 patients, associated with epithelial erosion. Two cases in the COVID-19 group (case #2 and #4) showed evident lymphocytic vasculitis of small subepithelial vessels associated with foci of coagulative necrosis (Figure 1), while granulomas and granulation tissue were not observed in any patient. Squamous metaplasia was identified in patients with a longer duration of intubation (patient #1 and #2). Acute tracheitis was not identified in any tracheal sample. Inflammatory cells, as lymphocyte and macrophages, were detected by immunohistochemical staining in each COVID-patient sample. CD20, CD3, CD4, and CD8 lymphocyte and CD68 macrophages cells were counted in 10 different randomly chosen areas in each processed slide with 40× magnification. Counting was performed blindly by two independent observers.

### Detection of the SARS-CoV-2

The SARS-CoV-2 was detected in one out of four COVID-19 samples (case #4) by the RT-PCR assay whereas all tracheal samples were negative at immunohistochemical detection with anti-SARS Nucleocapsid Protein.

### COVID-19 Samples Have a Remarkable Gene Expression Alteration

**Figure 2:**
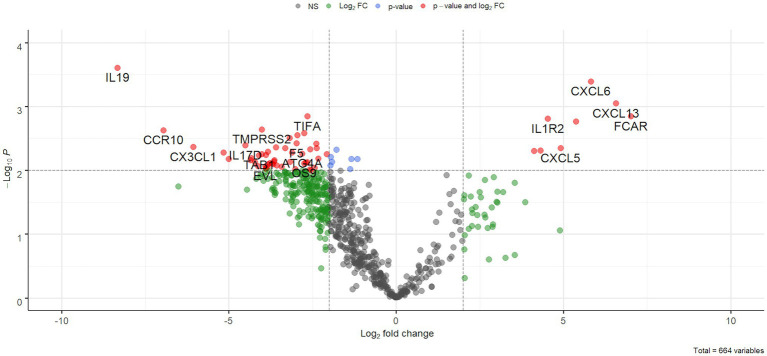
Volcano plot. Results of differential expression analysis were plotted as fold changes (*x*-axis) and -log10 of value of ps (*y*-axis). Dotted lines represent a value of *p* of 0.01 (horizontal line) and a 2-fold absolute value (vertical lines).

After filtering out low count genes, 664 transcripts were considered for further analyses. Compared to the control group, COVID-19 samples showed marked gene expression changes with a trend toward gene down-regulation. In fact, a statistically significant difference was identified in 332 out of 664 genes (Figure 2) but, when adjusting for multiple comparisons, no genes had a false discovery rate (FDR) less than 0.05 due to the low statistical power.

**Figure 3:**
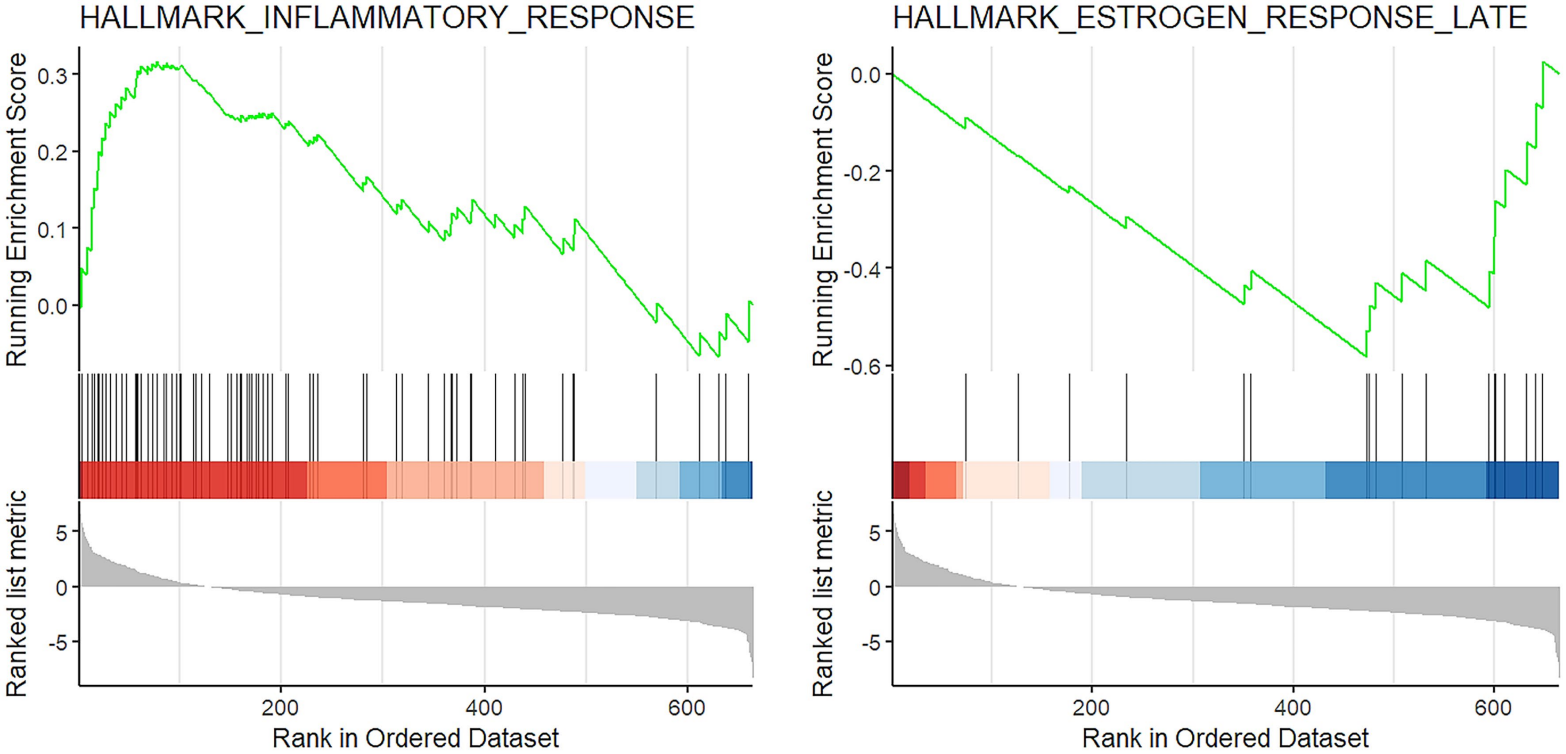
GSEA results. Enrichment plot of the two significantly altered gene sets in COVID-19 cases: activation of inflammatory response (left) and suppression of late estrogen response (right).

The GSEA showed that two gene sets were significantly deregulated in COVID-19 patients. In details, the HALLMARK_INFLAMMATORY_RESPONSE was activated (FDR = 0.0001, normalized enrichment score (NES) =1.91) and the HALLMARK_ESTROGEN_RESPONSE_LATE was suppressed (FDR = 0.05, NES = −1.82; Figure 3).

**Figure 4:**
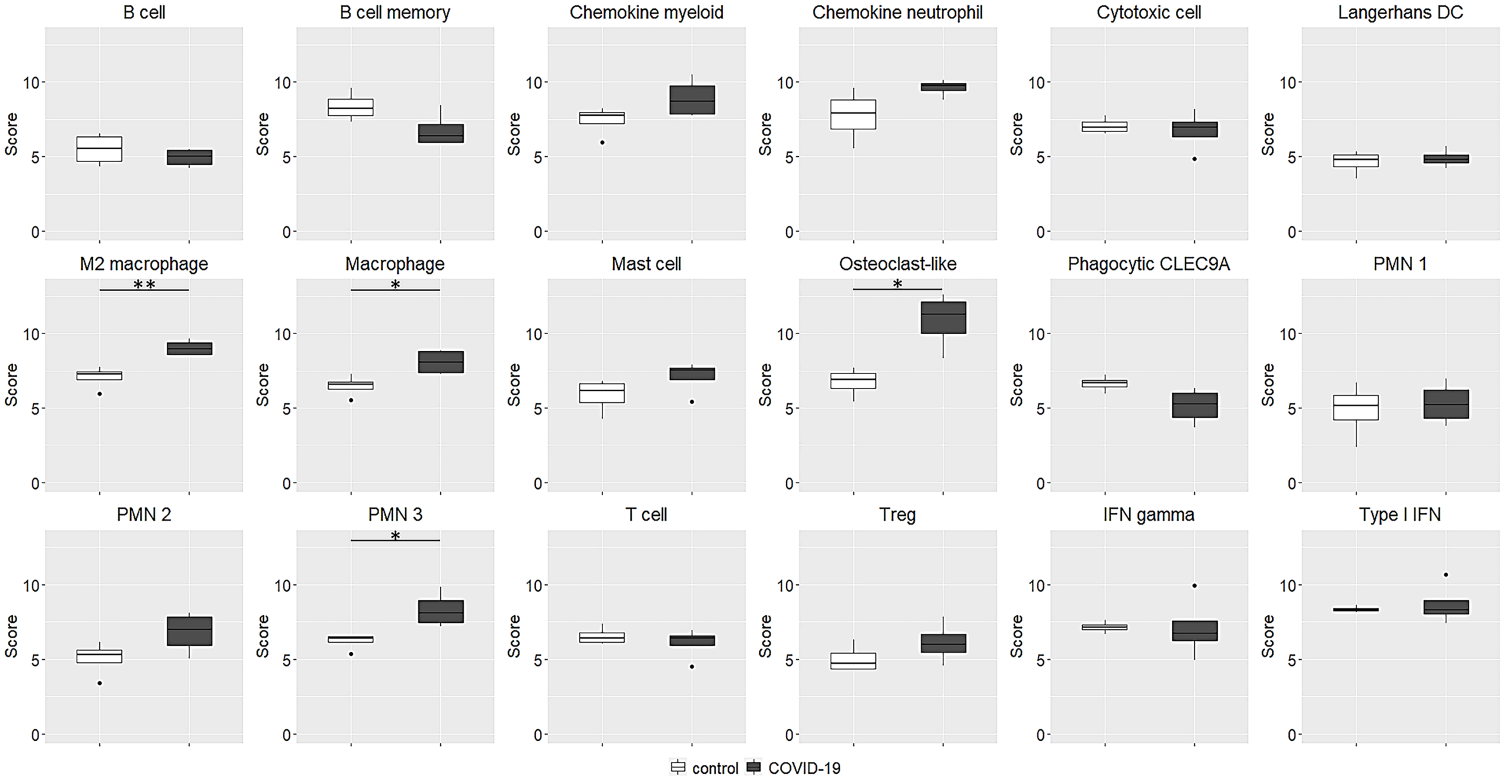
Immune cell abundance estimated by transcriptional data. The abundances are expressed as log2 expression. Macrophage, M2 macrophage, osteoclast-like and polymorphonuclear neutrophils scores were significantly higher in COVID-19 tracheal samples. ^*^p < 0.05; ^**^p < 0.01. PMN, polymorphonuclear neutrophils; IFN, interferon.

**Figure 5:**
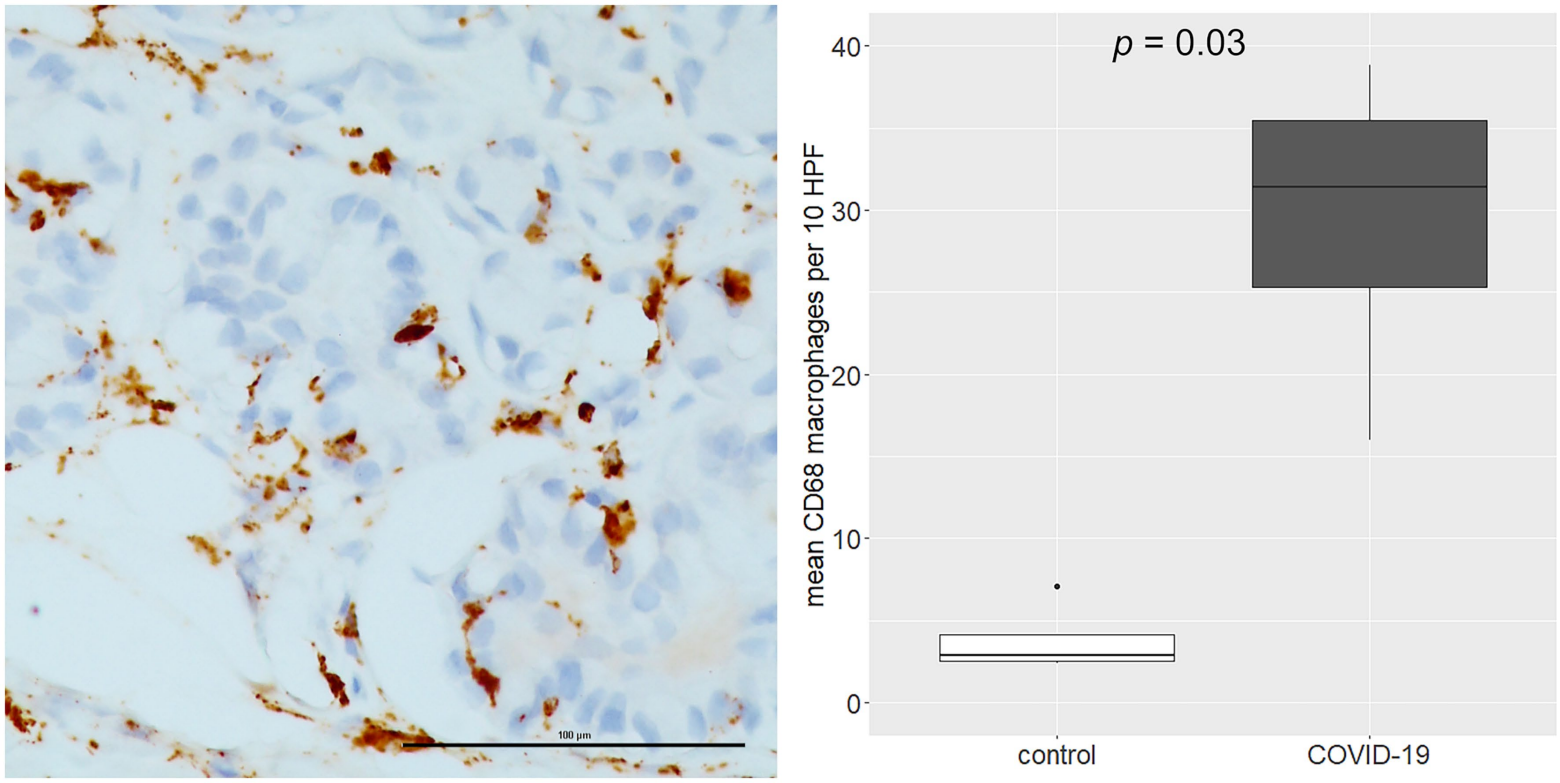
Immunohistochemistry (IHC) analysis of CD68 macrophages (left side). The over-representation of CD68 macrophages was confirmed by IHC analysis (right side). Scale bar refers to 100 μm.

As regards immune cell abundances, COVID-19 tracheal samples showed higher scores of innate immune cells including macrophages (*p* = 0.03), M2 macrophages (*p* = 0.007), osteoclast-like (*p* = 0.01) and polymorphonuclear neutrophils (*p* = 0.03; Figure 4). These results were confirmed by the immunohistochemistry (IHC) analysis; in fact, CD68 macrophages were significantly more abundant in the COVID-19 samples than in samples of the control group (*p* = 0.03; Figure 5).

## Discussion

Many papers related to acute and late laryngotracheal complications in COVID-19 patients subjected to invasive mechanical ventilation have been published in the last few months (Alturk et al., 2021; Balakrishnan et al., 2021; Fiacchini et al., 2021; Mahmood et al., 2021; Sandu, 2021; Scholfield et al., 2021). In order to explain the increase in the incidence of this particular type of complication, several etiopathogenetic hypotheses have been proposed. However, none of these appear to be more acceptable than others and the cause is likely to be found in a combination of multiple triggers. Among them, the possibility that the laryngotracheal mucosa could be a site of high viral replication intrigued us and led us to investigate in this sense. This hypothesis was corroborated by the findings of Bradley et al. (2020). As a matter of fact, they identified viral particles in the tracheal epithelium of 12 autopsies. Moreover, other authors reported laryngotracheal oedema that might suggest virus-mediated inflammation (McGrath et al., 2020; Oliver et al., 2020; Osbeck Sandblom et al., 2021). For these reasons, the aim of this study was to identify the virus particles in the tracheal epithelium of live COVID-19 patients and to evaluate any histological and genetic differences compared to a control group.

Histologic evidence in COVID-19 tracheal biopsies were similar to those observed in other series, with lymphomonocytic subepithelial inflammation and vasculitis, with small foci of coagulative necrosis (Lucchi et al., 2020). However, in our small series, immunohistochemical detection of SARS-CoV-2 was negative, differently from other reports (Martines et al., 2020). This is probably due to the prolonged oro-tracheal intubation and the use of electrified instruments to perform the tracheostomy that both caused de-epithelialization of the tracheal samples. However, in one case the SARS-CoV-2 genome was detected at very low load. The possibility that positivity of PCR assays represents viral spreading *via* blood/lymphatic vessels rather than specific virus tropism, should also be considered.

COVID-19 samples showed an intense inflammatory response and a strong gene deregulation. In addition, these cases presented a greater infiltration of innate immune cells (mostly macrophages or cells with phagocytic activity), which are coherent with an early phase of immune response. It has been widely discussed that both in patients who eventually died of Severe Adult Respiratory Syndrome (SARS) and in animal models, extensive lung damage is associated with high initial viral loads, increased inflammatory monocytes/macrophages accumulation in the lungs and elevated serum proinflammatory cytokines (Nicholls et al., 2003; Perlman and Dandekar, 2005). While much is known about the terminal phase of SARS, little is known about the early immune events during the acute phase of infection.

Monocytes and macrophages may be directly infected by SARS-CoV-2 through ACE2-dependent process or indirectly infected *via* ACE2-independent pathways and phagocytosis of virus-containing apoptotic bodies. SARS-CoV-2 can effectively suppress the anti-viral IFN response in monocytes and macrophages. Upon infection, monocytes migrate to tissues where they become infected resident macrophages, allowing viruses to spread through all organs and tissues. The SARS-CoV-2-infected monocytes and macrophages can produce large amounts of numerous types of pro-inflammatory cytokines and chemokines, which contribute to the local tissue inflammation and dangerous systemic inflammatory response as named cytokine storm (Jafarzadeh et al., 2020).

Interestingly, studies comparing the host response to SARS-CoV-2 and influenza viruses in the upper respiratory tract, observed very low levels of virus but a robust transcriptional response with differential expression of transcripts implicated in two populations of immune cell signatures (Blanco-Melo et al., 2020). The first population included common markers for monocytes and lymphocytes, and the induction of these genes was comparable between SARS-CoV-2 and influenza virus. Consistent with this, they found significant induction of monocyte-associated chemokines such as CCL2 and CCL8. In addition, their data suggest that neutrophils could also contribute to the disease observed in COVID-19 patients, as demonstrated by CXCL2 and CXCL8 induction, differently from influenza virus infection. This is consistent with data showing elevated circulating neutrophil levels among COVID-19 patients (Chen et al., 2020; Qin et al., 2020), which may have prognostic value for identifying individuals at risk for developing severe disease. These data, obtained from the analysis of *in vitro* and *ex vivo* samples, support what we observed in our series, highlighting the multiplicity of disease pathways of SARS-CoV-2.

Herein, we observed also the suppression of estrogen response pathway. It was already reported that sex hormones, including estrogens, may regulate both innate and adaptive immune response (Khan and Ansar Ahmed, 2015). Consequently, estrogen suppression has been involved in autoimmune conditions (Moulton, 2018; Kim et al., 2019). It is interesting to note that, in support of this observation, the groups analyzed show a gender imbalance, with a preponderance of female sex in the COVID-19 group compared to the control group.

This work has several limitations. The first is the small sample size. As a matter of fact, the “tracheo-team” decided to perform an open surgical tracheostomy in only four patients (20%) due to the fact that the percutaneous technique has several advantages, especially in terms of aerosolization. The second is relative to its retrospective and single center nature. Third, we were not able to identify the viral genome in three tracheal samples due to the aforementioned reasons. For all these issues we were not able to draw any definitive conclusions. Anyway, we can take some ideas from these data, especially related to the gene expression alterations. Even without the identifications of SARS-CoV-2 genome and prominent histological differences between the two groups, the gene expression is clearly altered in the COVID-19 group meaning that a different inflammatory response is taking place in these patients. However, it must be considered that the control group was not matched for the main pathology that had caused the ICU hospitalization. In particular, no patient in the control group suffered from ARDS, so all of them may not have an ongoing inflammatory process in the respiratory tract. Certainly, other studies with larger and adequate sample size are needed to confirm these data, but this could be another piece in the composition of this difficult puzzle of laryngotracheal complications in COVID-19 patients.

In conclusion, we cannot confirm that the trachea is a site of high viral replication. However, the tissue samples of the COVID-19 group showed a significant alteration of gene expression in two gene sets (activation of the HALLMARK_INFLAMMATORY_RESPONSE and suppression of the HALLMARK_ESTROGEN_RESPONSE_LATE) compared to the control group, meaning that the inflammatory response of the COVID-19 patients is completely different. Further studies are warranted to investigate these aspects.

## Data Availability Statement

The original contributions presented in the study are included in the article/supplementary material, further inquiries can be directed to the corresponding authors.

## Ethics Statement

This study was approved by the Local Ethics Committee on June 24, 2021. Written informed consent to collect deidentified data was obtained from all patients. This study followed the Strengthening the Reporting of Observational Studies in Epidemiology (STROBE) reporting guideline.

## Author Contributions

All authors contributed to the study conception and design. Material preparation, data collection and analysis were performed by GiF, AP, AMP, and LB. The first draft of the manuscript was written by GiF and AP and all authors commented on previous versions of the manuscript. Review and editing were performed by GiF, AP, MP, ID, and LB. The methodology of study was supervised by GaF and FG. All authors read and approved the final manuscript.

## Conflict of Interest

The authors declare that the research was conducted in the absence of any commercial or financial relationships that could be construed as a potential conflict of interest.

## Publisher’s Note

All claims expressed in this article are solely those of the authors and do not necessarily represent those of their affiliated organizations, or those of the publisher, the editors and the reviewers. Any product that may be evaluated in this article, or claim that may be made by its manufacturer, is not guaranteed or endorsed by the publisher.
